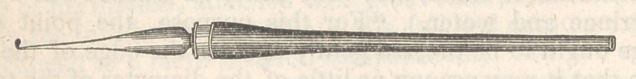# On the Removal of Foreign Bodies from the Meatus Auditorius Externus

**Published:** 1845-04

**Authors:** James Bryan


*On the removal of Foreign Bodies from the Meatus Auditorius Externus.* By James Bryan, M. D.

The frequency with which accidents of this kind occur in the practice of every physician makes it a matter of considerable importance. The bent form of the passage, with the great sensibility of the part, particularly when irritated and inflamed by the presence of a foreign body, present great difficulties in the way of its extraction.

A collection of the ordinary cerumen of the canal, producing not unfrequently considerable deafness, and accompanied with various noises in the ear, may generally be brought away by means of the ordinary ear-scoop or curette, followed by the judicious application of pure warm water. Water, according to the experiments of Haygarth, is the best solvent of the ear wax, and ought, consequently, in all these cases to be used in preference to the various oils and other substances sometimes recommended.

When ants, caterpillars, spiders or other insects creep into the meatus, the introduction of a little olive oil will be proper, which will have the effect either to destroy them, or by filling their spiracula, cause them to come out voluntarily.

When dead, if small, they may easily be washed out with an ear-syringe and water. "For this purpose, the point of the syringe ought to be pressed gently against the edge of the meatus, so that it may occupy as little of the diameter of the tube as possible, and when the injection arrives at the membrana tympani, the regurgitation will force the body upwards."* Should the insect, however, be too large for this, it may in most cases be taken out without much trouble by means of a pair of small forceps.

The greatest difficulty will always be found in the extraction of more solid bodies, such as beans, peas, cherry-pits, pieces of glass, lead, iron, and similar substances. The, evils which the protracted retention of such articles in the meatus entail, are inflammation of the lining membrane and neighbouring tissues—ulceration of the membrana tympani, destruction of the ossicula auris, and purulent discharges, involving the temporal bone itself, and even the membranes or substance of the brain.

The following case reported by Fabricius Hildanus, and quoted by Samuel Cooper, will illustrate some extraordinary symptoms resulting from this accident:

"After four surgeons, who had been successively consulted, had in vain exerted all their industry to extract a bit of glass from the left ear of a young girl, the patient found herself abandoned to the most excruciating pain, which soon extended to all the side of the head, and which, after a considerable time, was followed by a paralysis of the left side, a dry cough, suppression of the menses, epileptic convulsions, and, at length, an *atrophy of the left arm*." She was cured by the extraction of the piece of glass, which had been in her ear eight years."

*Buchanan.

Cherry-pits, grains of corn, beans and other substances, which expand on being exposed to the fluids of the part, in addition to the irritation induced by their presence, become embedded in the tissues, and in this condition are very difficult to remove. I have found it impossible to succeed with the forceps or other instruments ordinarily used, and the operation recommended by some of the old aurists, of cutting down upon the meatus *behind* the auricle, makes the matter no better, and is consequently very properly entirely abandoned by the profession.

I have been enabled, by means of an instrument used by dentists for the purpose of cleansing the cavities of decayed teeth, to extract cherry-pits and grains of corn, which have baffled the skill of some of our most eminent surgeons. The instrument is about four inches long, and will be sufficiently explained by reference to the accompanying cut.

The patient sits upon a chair, and an assistant supports his head, turned to one side, throwing if possible a strong light upon the ear affected. The instrument is carefully introduced, and pushed forwards until it reaches the substance, when keeping the curved edge towards the axis of the meatus, it is rapidly passed beyond the body, between it and the wall of the passage. As soon as this is done, the surgeon will find that he has complete control over it, and by traction can easily dislodge it from its bed. A repetition of the operation is much easier, both to the surgeon and the patient, and will in most cases be sufficient to extract the foreign body.

The previous introduction of some bland oil, as recommended by Velpeau, for the purpose of allaying irritation and causing the foreign substance to glide over the surface with greater facility, may be of use—but I have not found it of much importance. After the operation, the introduction into the meatus of some weak lead water may be necessary, or even the establishment of counter-irritation behind the ear or on the nape of the neck.

## Figures and Tables

**Figure f1:**